# Sotatercept as an Add-On to Background Therapy in Idiopathic Pulmonary Arterial Hypertension: Insights from a Real-World Cohort

**DOI:** 10.3390/ph19050760

**Published:** 2026-05-13

**Authors:** Charalampos Filippatos, Ioannis Boutsikos, Nikolaos-Iason Tepetes, Andrew Xanthopoulos, Ernesto Ruiz Duque, Rabea Asleh, George Giannakoulas, Alexandros Briasoulis

**Affiliations:** 1 Department of Clinical Therapeutics, National and Kapodistrian University of Athens, 11528 Athens, Greece; 2 Department of Cardiology, University Hospital of Larissa, University of Thessaly, 41110 Larissa, Greece; 3 Division of Cardiovascular Medicine, University of Iowa, Iowa City, IA 52242, USA; 4 Heart Institute, Hadassah University Medical Center, Hebrew University of Jerusalem, Jerusalem 9190500, Israel; 5 1st Department of Cardiology, School of Medicine, Faculty of Health Sciences, Aristotle University of Thessaloniki, 54124 Thessaloniki, Greece

**Keywords:** idiopathic, pulmonary arterial hypertension, PAH, sotatercept, background therapy

## Abstract

**Background**: Sotatercept, a first-in-class activin-signaling inhibitor, demonstrated efficacy in pulmonary arterial hypertension (PAH) in the pivotal PULSAR and STELLAR trials. However, whether these benefits and the drug’s distinct safety profile translate to routine clinical practice in the treatment of idiopathic PAH remains unclear. **Methods**: We conducted a retrospective study using the TriNetX global health research network. Adult patients with idiopathic PAH treated with standard-of-care (SoC) background therapies were identified. Those receiving add-on sotatercept were matched 1:1 with patients on background therapy alone using propensity score matching. **Results**: A total of 1378 matched patients (689 per arm) were included. The median follow-up was 13.8 months for the SoC group and 10.3 months for the sotatercept group; therefore, the analysis was limited to up to 12 months. Sotatercept was associated with a 49% reduction in the risk of all-cause mortality (HR = 0.51, *p* = 0.002). A significant reduction was observed in all-cause hospitalizations for the sotatercept arm (HR = 0.44; 12-month rate 6.3% vs. 11.5%, *p* = 0.010). Safety analysis revealed 2.5-fold increased odds of epistaxis (OR = 2.68, *p* < 0.001) and 4-fold increased odds of erythrocytosis (OR = 4.26, *p* < 0.001) with sotatercept, while thrombocytopenia rates were similar (OR = 0.98, *p* = 0.946). Sotatercept seems to be associated with a higher risk of hypertension compared to background therapy alone (OR = 1.85, *p* = 0.155). **Conclusions**: In this large real-world cohort, the addition of sotatercept to SoC background therapy significantly improved survival in idiopathic PAH. While bleeding events, erythrocytosis and elevated blood pressure seemed to be more frequent with sotatercept, the overall safety profile was acceptable, suggesting that the substantial survival and hospitalization benefits outweigh these risks in routine clinical practice.

## 1. Introduction

Pulmonary arterial hypertension (PAH) is a progressive disease that leads to increased pulmonary artery (PA) pressure and subsequent right ventricular dysfunction [1]. Pathologically, the disease is driven by a complex interplay of multiple pathways. A key mechanism, particularly in idiopathic forms, involves a critical imbalance between bone morphogenetic protein receptor type 2-mediated anti-proliferative (BMPR2) signaling and activin receptor type IIA-mediated pro-proliferative signaling [2,3]. This dysregulation, along with the influence of other crucial mediators such as endothelin and prostaglandins, leads to endothelial dysfunction and smooth muscle cell hyperproliferation within the vessel wall that subsequently increases pulmonary vascular resistance [4]. While traditional therapies targeting endothelin, nitric oxide, and prostacyclin pathways provide essential vasodilation and have some impact on disease progression [5], they often fail to fully halt or reverse this multifactorial underlying vascular remodeling.

Sotatercept is a novel first-in-class activin-signaling inhibitor that acts as a ligand trap for pro-proliferative activins and related growth factors, thereby restoring antiproliferative balance [6]. The efficacy and safety of sotatercept were evaluated in two pivotal randomized, double-blind, placebo-controlled trials, PULSAR and STELLAR. In both studies, the sotatercept group had significantly lower pulmonary vascular resistance (PVR), as well as better 6 min walking distance (6MWD) and lower NT-proBNP levels [7,8].

However, randomized controlled trials (RCTs) are conducted under idealized conditions, often excluding patients with significant comorbidities or complex background regimens. Consequently, whether the profound efficacy and distinct safety profile of sotatercept—specifically regarding bleeding risks and hemoglobin elevation [9]—translate to routine clinical practice remains a crucial unanswered question.

This study aims to compare the clinical outcomes of sotatercept as an add-on therapy in patients with idiopathic PAH who receive the standard of care, with a specific focus on survival and the evaluation of key safety events such as epistaxis, thrombocytopenia, erythrocytosis and elevated blood pressure.

## 2. Results

### 2.1. Study Population and Follow-Up

A total of 5539 patients diagnosed with idiopathic PAH on or after 2024 receiving standard of care background PAH therapy and 689 patients receiving sotatercept as add-on therapy, were identified. After 1:1 propensity score matching, 689 patients were included in each cohort, yielding well-balanced groups across all covariates included. The median follow-up duration was 13.8 months in the standard-of-care cohort and 10.3 months in the sotatercept add-on cohort. Propensity score matching successfully balanced all demographic, comorbidity, medication and laboratory covariates, while residual numerical imbalances were within an acceptable range (standardized mean differences, SMD < 0.1). Furthermore, visual inspection of the propensity score density plots further confirms the efficacy of the matching algorithm, as post-matching distributions demonstrated a high degree of overlap and congruence (Supplementary Materials, Figure S1). Baseline patient characteristics which accounted for cohort matching are presented in Table 1.

### 2.2. Efficacy Outcomes

All primary outcomes were analyzed up to the 1-year timepoint to account for the follow-up discrepancy and to ensure the reliability of KM estimates.

During the 1-year follow-up period, a total of 60 (8.7%) and 29 (4.2%) patients died in the no add-on and add-on arms, respectively. Patients that received sotatercept as an add-on to background therapy demonstrated a 49% reduced risk of death [HR = 0.51, 95% CI: 0.33–0.79, *p* = 0.002], compared to those who did not. The corresponding overall survival (OS) rates at 6 months and 12 months were 93.5% and 96.1%, and 89.8% and 94.5%, respectively.

Furthermore, among 357 and 292 evaluable patients in the no add-on and add-on arms, there were 36 (10.1%) and 12 (4.1%) cases of any-cause hospitalizations, respectively. Crude risks analysis revealed a significant reduction in the sotatercept arm (RR = 0.41, 95% CI: 0.22–0.77, *p* = 0.004) which was further validated in the time-to-event analysis (12-month rate 6.3% vs. 11.5%), as they demonstrated a 56% reduced risk of hospitalization (HR = 0.44, 95% CI: 0.23–0.84, *p* = 0.010).

Among 185 evaluable patients in the non-add-on arm and 107 in the add-on arm, there were 31 (16.8%) and 25 (23.4%) new cases of heart failure (HF), respectively. No significant difference in crude risks was observed (RR = 1.39, 95% CI: 0.87–2.23), which was further confirmed in the time-to-event analysis (HR = 1.57, 95%CI: 0.92–2.67, *p* = 0.090; 12-month rates 21.0% vs. 32.8%) (Supplementary Materials, Figure S2).

No patients in either arm underwent lung transplantation during the 12-month follow-up.

### 2.3. Safety Outcomes

No differences were observed regarding thrombocytopenia, as 32 (6.0%) and 30 (5.9%) of patients in the no add-on and add-on arms developed it, respectively (OR = 0.98, 95% CI: 0.59–1.64, *p* = 0.946). There was a notable increase in epistaxis events in the sotatercept arm (*n* = 43, 7.2%) compared to the no add-on arm (*n* = 17, 2.8%), which translates to a 2.5-fold increase in odds (OR = 2.68, 95% CI: 1.51–4.76, *p* < 0.001). Hypertension rates seemed to be higher in the sotatercept (*n* = 16, 35.6%) arm compared to those who did not receive the drug (*n* = 14, 23.0%), but results were non-significant (OR = 1.85, 95% CI: 0.85–2.84, *p* = 0.155). Erythrocytosis was observed in more patients in the sotatercept arm (13.6% vs. 3.6%; OR = 4.26, 95%CI: 2.52–7.20, *p* < 0.001). The patient count for the adverse events of secondary polycythemia and diseases of the capillaries was too small (<10), so detailed results were not displayed by TriNetX’s built-in Analytics (Table 2).

**Figure 1:**
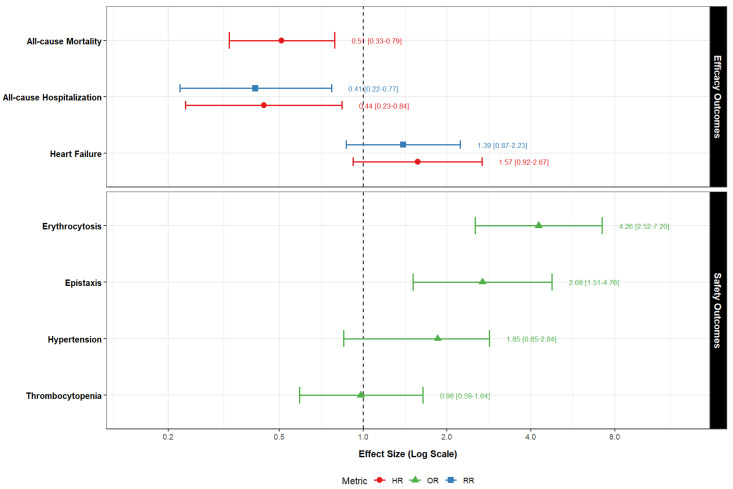
Forest plot of efficacy and safety effect estimates.

The aforementioned effect estimates for the efficacy and safety outcomes are portrayed in Figure 1.

## 3. Discussion

Our analysis showed that the real-world use of sotatercept on top of the approved PAH therapies had a markedly lower risk of death compared to non-use. This finding is in line with the results of Phase 2 and 3 PULSAR and STELLAR trials [7,8,9,10]. Sotatercept has a unique mechanism and hemodynamic benefit that differs from the alternative PAH therapies. It leads to a significant decrease in pulmonary vascular resistance, indicating partial reverse remodeling of the pulmonary arteries [11] and critical improvement in pulmonary artery compliance and elastance [12]. This reflects improved pulmonary artery distensibility, reduced right ventricular afterload and low right ventricular energy expenditure [12]. However, the absence of a pronounced increase on cardiac output is a landmark effect of sotatercept and a distinguishing feature compared with the currently approved therapies, which lead to reflex increase in cardiac output due to their systemic vasodilatory effects [13].

Data from the STELLAR trial [8] showed that systolic blood pressure and systemic vascular resistance increased after the use of sotatercept, leading to a small reduction in left ventricular ejection fraction. In our analysis, no difference in the risk of incident HF was observed between the two groups. In the Phase 2 PULSAR trial, improvements in objective measures of exercise capacity and cardiovascular stress (6 min walking distance and NT-proBNP) were also observed [7]. Improved right ventricular size and function in addition to RV–PA coupling indicate improved function of the cardiopulmonary circuit [14]. While negative myocardial repair was observed in an early zebrafish model [15], more recent preclinical studies using sotatercept analogs in mammalian models have demonstrated positive cardiopulmonary reverse remodeling [16]. This aligns with clinical data showing no deterioration of left ventricular function, which can also be predominantly explained by reduced pulmonary vasculature pressure [8].

Another safety issue that is raised with the use of sotatercept is its newly emerging and still incompletely characterized side effects. Sotatercept exerts its biological effects through modulation of the activin–growth differentiation factor (GDF) signaling axis, a pathway that plays a central role not only in pulmonary vascular remodeling but also in hematopoiesis, angiogenesis, and endothelial homeostasis. As such, the adverse effects observed with sotatercept are mechanistically predictable and warrant careful interpretation. More specifically, various studies have shown that sotatercept may provoke a reduction in the absolute number of platelets leading to hemorrhagic events.

In pivotal randomized trials and pooled analyses, the most frequently reported adverse events associated with sotatercept included thrombocytopenia, increased hemoglobin levels, epistaxis, telangiectasias, and cutaneous vascular abnormalities [7,8,9]. These findings reflect the systemic consequences of inhibiting activin signaling, which normally acts as a negative regulator of erythropoiesis and megakaryocyte maturation. By neutralizing circulating activins, sotatercept shifts the hematopoietic balance toward enhanced erythroid proliferation, predisposing patients to secondary polycythemia and, paradoxically, platelet count reductions. This dual effect may increase blood viscosity while simultaneously impairing primary hemostasis, thereby explaining the observed spectrum of bleeding manifestations [17].

In the present real-world analysis, patients treated with sotatercept as add-on to background therapy had a statistically significant 2.5-fold increase in the odds of epistaxis and a 4-fold increase in the odds of erythrocytosis, compared to plain background therapy. This observation coincides with the significantly increased rates of epistaxis and bleeding events observed in the STELLAR trial, compared to the placebo arm [8,10]. On the other hand, no differences were observed in the rates and subsequent odds of thrombocytopenia (OR = 0.98, *p* = 0.946). While data from the STELLAR and PULSAR trials showed an increase in thrombocytopenia events in the sotatercept arms, the absolute counts were small to allow for robust statistical conclusions. Another documented AE of treatment with sotatercept in the STELLAR trial is the observation of increased blood pressure in patients in the experimental arm [8]. In our real-world cohort, a similar but not significant trend was observed (35.6% vs. 23.0%, OR = 1.85, *p* = 0.155). Nevertheless, the relatively short follow-up duration, especially in the sotatercept cohort, limits definitive conclusions regarding long-term safety, particularly with respect to sustained erythrocytosis, vascular remodeling outside the pulmonary circulation, and potential thrombotic complications.

The primary strength of this study lies in its provision of large-scale real-world evidence that complements the findings of pivotal randomized controlled trials. While the STELLAR and PULSAR trials established the efficacy of sotatercept in controlled settings, our analysis of 1378 propensity-matched patients validates these benefits in a diverse, routine clinical practice population. Furthermore, the rigorous and successful employment of 1:1 propensity score matching allowed us to balance cohorts not just on demographics, but on critical clinical variables often omitted in observational studies, including specific prostacyclin therapies (treprostinil, epoprostenol), pulmonary stress testing history, and baseline cardiac biomarkers. This granular matching minimizes selection bias and strengthens the association observed between sotatercept and improved survival.

Despite the aforementioned strengths, several limitations must be noted. First, despite robust propensity score matching, the potential for residual confounding from unmeasured variables remains, preventing the establishment of definitive causal relationships. This is an inherent limitation of our study’s retrospective and real-world design. Second, the reliance on ICD-10 and RxNorm codes for case identification and outcome tracking introduces the possibility of misclassification bias or underreporting of the outcomes studied. Third, the median follow-up duration for the sotatercept cohort was relatively short (10.3 months), thus restricting our ability to fully characterize long-term safety risks related to complications that may require longer exposure to manifest. Moreover, the short follow-up duration also limited our analysis up to the 1-year timepoint, which may not be enough for safe conclusions regarding overall survival. Furthermore, the inherent constraints of retrospective EHR analyses restricted our ability to dynamically track longitudinal therapeutic escalation or mid-study adjustments to background PAH regimens; thus, potential residual confounding from subsequent treatment modifications cannot be fully excluded. Finally, the low absolute event counts or the low number of evaluable patients for specific safety outcomes, such as secondary polycythemia, diseases of the capillaries and hypertension, limited our statistical analysis, highlighting the need for larger, longer-term registry studies to fully define these risks.

In conclusion, the findings of this real-world analysis of 1378 patients support the significant benefits of sotatercept as an add-on therapy in PAH when used in appropriately selected patients and under structured monitoring protocols. Regular assessment of hemoglobin, platelet counts, and blood pressure should be considered essential components of sotatercept therapy, particularly during the early phases of treatment escalation. Future studies with longer follow-up and granular laboratory data will be essential to define optimal monitoring intervals, dose adjustment strategies, and patient populations at heightened risk for adverse effects.

## 4. Materials and Methods

### 4.1. Study Design

This retrospective, observational cohort study utilized real-world data from the TriNetX global health research network. Adult patients with idiopathic PAH who were treated with standard-of-care regimens (sildenafil, tadalafil, macitentan, ambrisentan, bosentan or selexipag) and sotatercept as an add-on were retrospectively enrolled in this study. To ensure the isolation of sotatercept’s drug effect, a control group was formed with patients treated with the same standard-of-care drugs but who did not receive sotatercept at any point during their follow-up. Εligible patients for the control arm included individuals diagnosed in or after 2024 to ensure contemporary PAH management.

### 4.2. Population-Defining ICD-10, ATC and RxNorm Codes

The ICD-10 code used to define the population’s condition (idiopathic pulmonary arterial hypertension) is the following: I27.0. The RxNorm codes used to define background therapies are: 136411 (sildenafil), 358263 (tadalafil), 1442132 (macitentan), 358274 (ambrisentan), 75207 (bosentan), 1729002 (selexipag). The RxNorm code used to define sotatercept as add-on therapy was 2678930.

### 4.3. Cohort Matching

All cohort pairs were matched using TriNetX’s built-in 1:1 propensity score matching algorithm. Matching was performed based on demographic characteristics (age, sex, race, and ethnicity) and clinical measurements including body mass index, systolic and diastolic blood pressure, and respiratory rate. The cohorts were also balanced on key laboratory parameters: hematocrit, platelets, hemoglobin, alanine aminotransferase, aspartate aminotransferase, total bilirubin, N-terminal pro-brain natriuretic peptide, and brain natriuretic peptide.

Additionally, matching included relevant comorbidities and historical factors such as sleep apnea, shortness of breath, essential hypertension, nicotine dependence, diastolic heart failure, thyroid disorders, interstitial and other pulmonary diseases, atherosclerotic heart disease, liver disease, chronic kidney disease (CKD), atrial fibrillation, diabetes mellitus, malaise and fatigue, and the presence of cardiac/vascular implants. Utilization of respiratory tract medications (including treprostinil and epoprostenol), anticoagulants and a history of pulmonary stress testing were also included in the matching model.

### 4.4. Index Event and Date

The index event, defined with the TriNetX Analytics tools (https://live.trinetx.com/), is the point in time when each patient in the cohort enters the analysis. The index date for each patient was defined as the first recorded diagnosis of idiopathic PAH or the first prescription of sotatercept/background therapy—whichever occurred later.

### 4.5. Outcomes

The primary efficacy outcomes included: overall survival, any-cause hospitalization and heart failure. Secondary, safety outcomes assessed included thrombocytopenia, epistaxis, erythrocytosis, hypertension, secondary polycythemia and diseases of the capillaries.

### 4.6. Statistical Analysis

Matching quality was assessed visually via density plots and quantitatively using SMDs, with SMD < 0.10 indicating excellent balance. All statistical analyses were conducted within the TriNetX Analytics platform. Baseline characteristics were summarized using medians and IQRs for continuous variables and frequencies with percentages for categorical variables. PSM was applied to control for potential confounding by balancing covariates between treatment cohorts. Time-to-event outcomes were analyzed using the Kaplan–Meier method, and differences between groups were assessed with log-rank tests. Hazard ratios (HR) and 95% confidence intervals were estimated using the Cox proportional hazards model. The proportional hazards assumption was evaluated for each Cox model using the Schoenfeld residuals test, reported by the Analytics platform. Crude risks and odds analyses involved risk ratios (RR) and odds ratios (OR). All effect estimates were calculated strictly among evaluable patients. All tests were two-sided, with *p* < 0.05 denoting statistical significance.

## Figures and Tables

**Table 1:** Baseline patient characteristics.

Variable	No Add-On (*n* = 689)	Sotatercept Add-On (*n* = 689)	SMD
Age (years)	58 (24)	57 (24)	0.007
Female—*n* (%)	519 (75.3%)	519 (75.3%)	<0.001
Not Hispanic or Latino—*n* (%)	507 (73.6%)	515 (74.7%)	0.027
White—*n* (%)	487 (70.7%)	491 (71.3%)	0.013
Hematocrit—*n* (%)	41.4 (9.4)	40.0 (8.0)	0.099
Platelets (×10^3^/μL)	212 (109)	209 (95)	0.071
Hemoglobin (g/dL)	13.0 (3.1)	13.5 (3.3)	0.095
BMI (kg/m^2^)	28.4 (9.7)	28.3 (10.3)	0.009
ALT (U/L)	15.0 (13.0)	15.0 (11.0)	0.063
AST (U/L)	20.0 (10.0)	19.0 (10)	0.098
Bilirubin (mg/dL)	0.6 (0.4)	0.5 (0.4)	0.096
Diastolic BP (mmHg)	67 (16)	68 (16)	0.013
Systolic BP (mmHg)	111 (21)	111 (23)	0.018
Respiratory rate (breaths/min)	17.0 (2.0)	18.0 (2.0)	0.064
NT-ProBNP (pg/mL)	479.0 (1715.0)	454.0 (1110.0)	0.011
Sleep apnea—*n* (%)	244 (35.4%)	234 (34.0%)	0.031
Shortness of breath—*n* (%)	221 (32.1%)	215 (31.2%)	0.019
Essential hypertension—*n* (%)	178 (25.8%)	178 (25.8%)	<0.001
HFpEF—*n* (%)	135 (19.6%)	131 (19.0%)	0.015
Disorders of thyroid gland—*n* (%)	100 (14.5%)	105 (15.2%)	0.020
CAD—*n* (%)	102 (14.8%)	100 (14.5%)	0.008
Diseases of liver—*n* (%)	97 (14.1%)	98 (14.2%)	0.004
CKD—*n* (%)	110 (16.0%)	120 (17.6%)	0.043
AF/Flutter—*n* (%)	93 (13.5%)	102 (14.8%)	0.037
DM—*n* (%)	89 (12.9%)	96 (13.9%)	0.029
ILD—*n* (%)	100 (14.5%)	79 (11.5%)	0.091
Other ILD—*n* (%)	118 (17.1%)	97 (14.1%)	0.084
COPD—*n* (%)	86 (12.8%)	100 (14.5%)	0.059
Malaise and fatigue—*n* (%)	57 (8.3%)	57 (8.3%)	<0.001
Cardiac, vascular implants and graft—*n* (%)	52 (7.6%)	56 (8.1%)	0.022
Smoking/nicotine dependence—*n* (%)	142 (20.6%)	144 (20.9%)	0.007
Respiratory tract medications—*n* (%)	385 (55.9%)	381 (55.3%)	0.012
Treprostinil—*n* (%)	190 (27.6%)	187 (27.1%)	0.010
Epoprostenol—*n* (%)	24 (3.5%)	22 (3.2%)	0.016
Anticoagulants—*n* (%)	273 (39.6%)	267 (38.8%)	0.018
Pulmonary stress testing—*n* (%)	247 (35.6%)	244 (35.4%)	0.009

Note: Continuous variables are presented as median (IQR); SMD = standardized mean differences, ALT = alanine aminotransferase, AST = aspartate aminotransferase, HFpEF = diastolic heart failure, CAD = coronary artery disease, CKD = chronic kidney disease, AF = atrial fibrillation, DM = diabetes mellitus, ILD = interstitial lung disease, COPD = chronic obstructive pulmonary disease.

**Table 2:** Adverse events.

AE	Events/Evaluable (Sotatercept)	Events/Evaluable (No Sotatercept)	OR	*p*-Value
Thrombocytopenia	30/512 (5.9%)	32/537 (6.0%)	0.98	0.946
Epistaxis	43/594 (7.2%)	17/601 (2.8%)	2.68	<0.001
Hypertension	16/45 (35.6%)	14/61 (23.0%)	1.85	0.155
Erythrocytosis	67/493 (13.6%)	19/533 (3.6%)	4.26	<0.001
Secondary polycythemia	-	-	-	-
Diseases of the capillaries	-	-	-	-

## Data Availability

The data presented in this study are openly available in TriNetX global health research network platform at https://live.trinetx.com/.

## Supplementary Materials

The following supporting information can be downloaded at: https://www.mdpi.com/article/10.3390/ph19050760/s1. Figure S1: Propensity density score curves; Figure S2: All-cause mortality; Figure S3: Any-cause hospitalizations; Figure S4: Heart failure.

## Abbreviations

The following abbreviations are used in this manuscript:

6MWD	6 min walking distance
AE	Adverse event
AF	Atrial fibrillation
ALT	Alanine aminotransferase
AST	Aspartate aminotransferase
BMI	Body mass index
BMPR2	Bone morphogenetic protein receptor type 2
BP	Blood pressure
CAD	Coronary artery disease
CKD	Chronic kidney disease
COPD	Chronic obstructive pulmonary disease
DM	Diabetes mellitus
GDF	Growth differentiation factor
HF	Heart failure
HFpEF	Diastolic heart failure
HR	Hazard ratio
ILD	Interstitial lung disease
NT-proBNP	N-terminal pro-brain natriuretic peptide
OR	Odds ratio
OS	Overall survival
PA	Pulmonary artery
PAH	Pulmonary arterial hypertension
PSM	Propensity score matching
PVR	Pulmonary vascular resistance
RCTs	Randomized controlled trials
ΡΡ	Relative risk/Risk ratio
SMD	Standardized mean differences
SoC	Standard of care
