# Supplementary material for: Sotatercept as an Add-On to Background Therapy in Idiopathic Pulmonary Arterial Hypertension: Insights from a Real-World Cohort
Source: Pharmaceuticals (Basel). 2026 May 13;19(5):760. doi: 10.3390/ph19050760 (PMC13209685; doi:10.3390/ph19050760)
Supplement: Supplementary file 1 [file pharmaceuticals-19-00760-s001.zip › pharmaceuticals-4309702-supplementary.pdf]

Supplementary Figure S1. Propensity density score curves

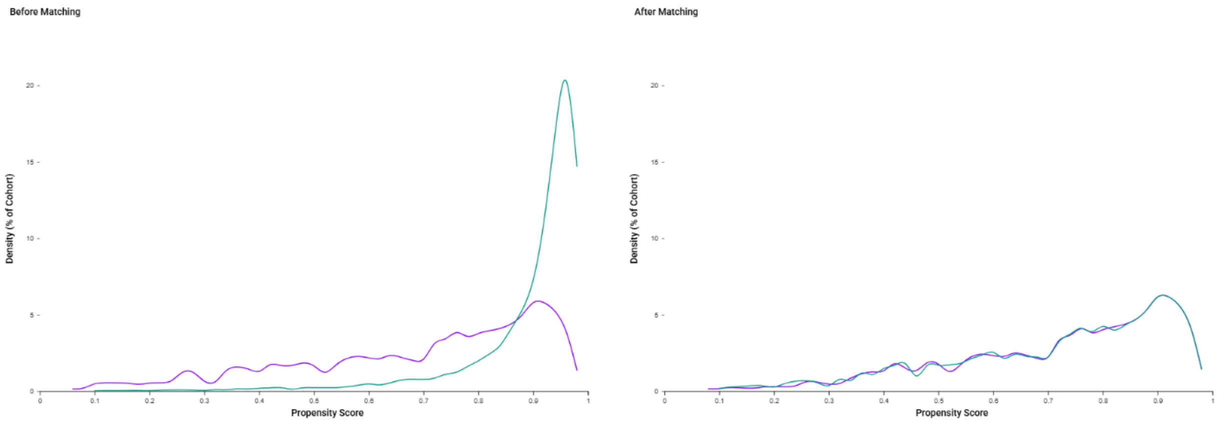

Note: Purple= Sotatercept add-on, Green= No sotatercept add-on

Supplementary Figure S2. All-cause mortality

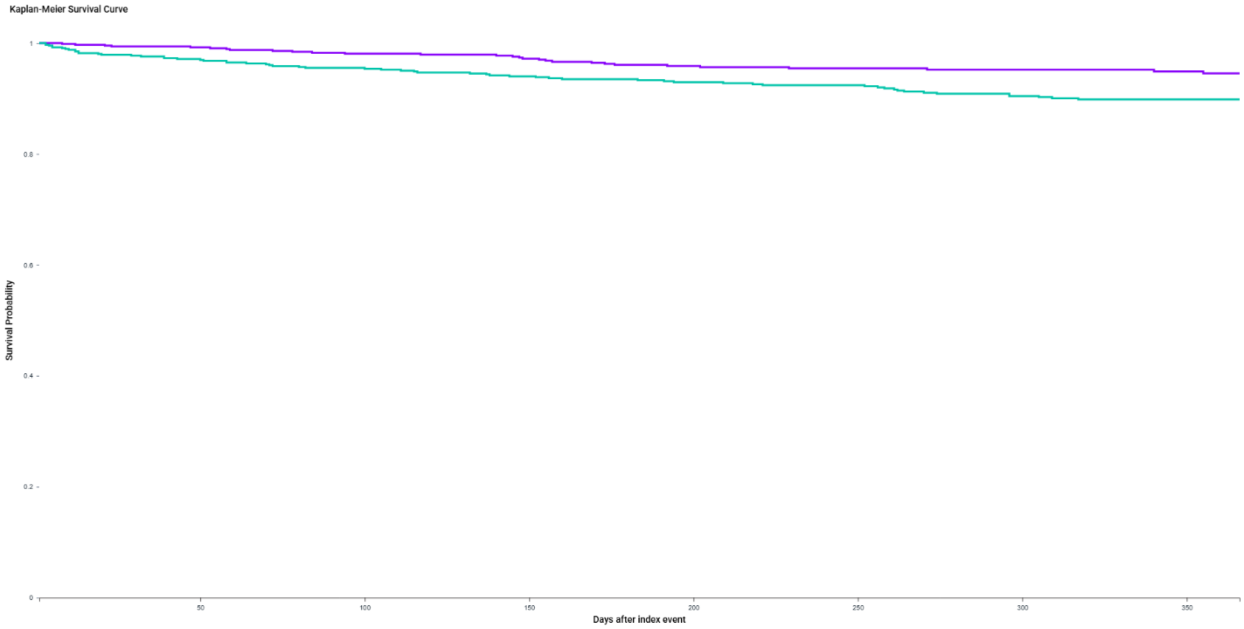

Note: Purple= Sotatercept add-on, Green= No sotatercept add-on

Supplementary Figure S3. Any-cause hospitalizations

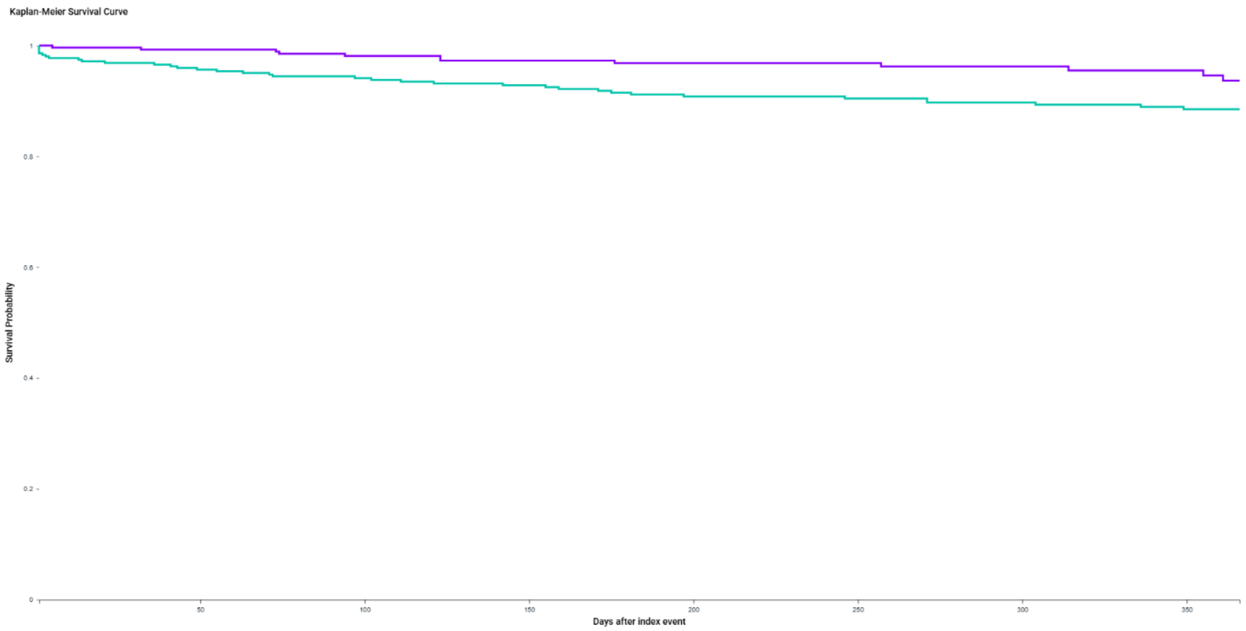

Note: Purple= Sotatercept add-on, Green= No sotatercept add-on

Supplementary Figure S4. Heart failure

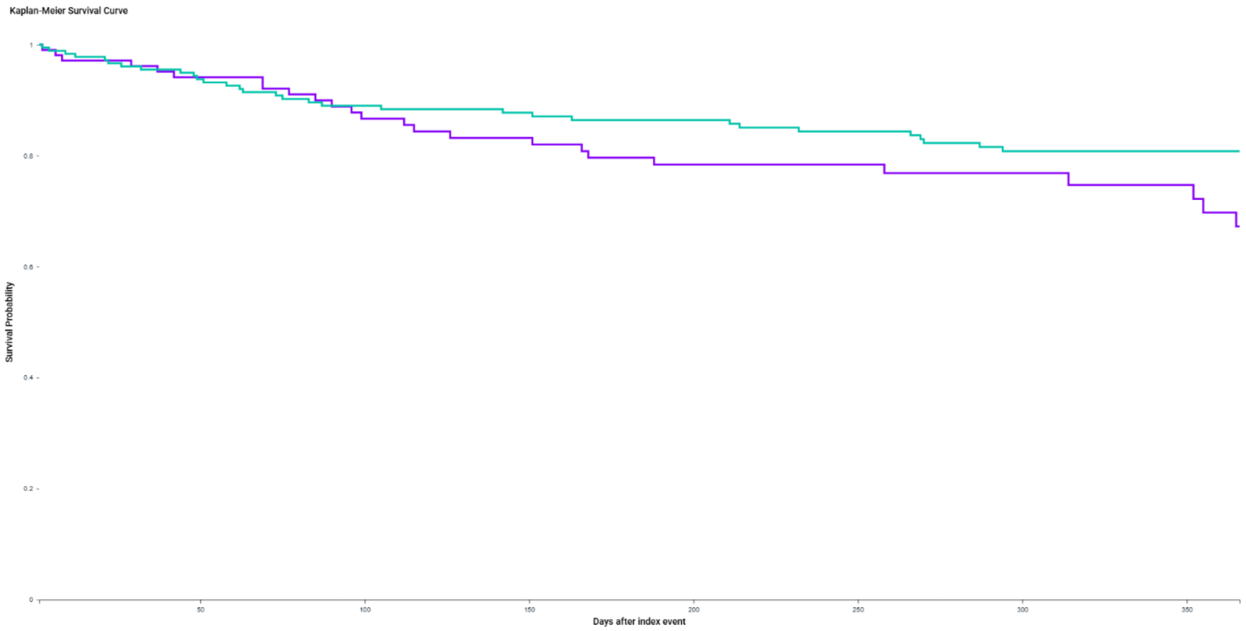

Note: Purple= Sotatercept add-on, Green= No sotatercept add-on
